# The protective effect and potential mechanisms of eugenol against *Salmonella* in vivo and in vitro

**DOI:** 10.1016/j.psj.2022.101801

**Published:** 2022-02-23

**Authors:** Xin Zhao, ShuMei Zheng, SiMin Wei, QiMing Tian, Ya Tao, RuoNan Bo, MingJiang Liu, JinGui Li

**Affiliations:** ⁎ School of Veterinary Medicine, Yangzhou University, Yangzhou 225009, PR China; † Jiangsu Co-innovation Center for Prevention and Control of Important Animal Infectious Diseases and Zoonoses, Yangzhou 225009, PR China; ‡ Joint International Research Laboratory of Agriculture and Agri-Product Safety, the Ministry of Education of China, Yangzhou University, Yangzhou, Jiangsu 225009, PR China

**Keywords:** eugenol, *Salmonella* Typhimurium, broiler, inflammatory response, intestinal tight junction

## Abstract

*Salmonella enterica* serovar Typhimurium (***S.* Typhimurium**) continues to be a serious concern to the poultry industry as a bacterial foodborne zoonosis, which generally results in intestinal inflammation and barrier dysfunction or even death. Eugenol is a phenolic compound with various pharmacological activities involved antioxidant, anti-inflammatory, and antibacterial effects, which is expected to be an effective nonantibiotic therapy. The purpose of this study was to explore the protective effects of eugenol in the cellular and broiler models of *S.* Typhimurium infection and the possible underlying mechanisms. The results of animal infection showed that eugenol treatments enhanced the relative weight gains and survival rates of broilers with a reduction of the organ bacterial load and intestinal ultrastructural injury. Moreover, eugenol significantly inhibited the mRNA levels of myeloid differentiation factor 88 (**MyD88**) and toll-like receptor-4 (**TLR4**), then declined the phosphorylation of p65 and IκBα of NF-κB pathway and the expressions of inflammatory factors (TNF-α, IL-1β, IL-2, and IL-18) in duodenum tissues, while maintained the expressions of intestinal tight junction proteins (ZO-1, claudin-1, occludin). Further experiments in vitro revealed that eugenol markedly inhibited the adhesion and invasion of *S.* Typhimurium to RAW264.7 or IEC-6 cells, then reduce bacterial multiplication in IEC-6 or DF-1 cells. In conclusion, eugenol could defend broilers from *S.* Typhimurium infection by stabilizing the intestinal mucosal barrier and relieving inflammatory response, as well as inhibiting bacterial adhesion and invasion to cells.

## INTRODUCTION

*Salmonella enterica* serovar Typhimurium (***S.* Typhimurium**), Gram-negative intracellular bacteria, is a global foodborne enteropathogen, which causes substantial economic losses in the poultry industry and public health security worldwide (Kumar et al., 2019). Once the hosts (such as avian, pig, and cattle) consume polluted water or food, the bacteria will colonize and invade intestinal epithelium to impair the intestinal barrier and induce enteric inflammation, triggering the diarrhea-predominant gastrointestinal disease, which ultimately affects the poultry production performance (Fàbrega and Vila, 2013; Horstmann et al., 2020). Tight junctions are the first defense line of the intestinal barrier against the invasion of microbial pathogens, then maintain the epithelial integrity and function (Sears, 2000; Vazquez-Terres et al., 1999). It has been shown that type III secretion system (**T3SS**) is centrally involved in the adhesion and invasion of *S*. Typhimurium to host cells, which cause intestinal tight junction (**TJ**) disruption and inflammation, as well as further lead to bacteria translocation through disrupted gut barrier (Liu et al., )*.* It is conceivable that this causes lipopolysaccharide (**LPS**) translocation, a component of Gram-negative bacteria (Yang et al., 2017). Then toll-like receptor-4 (**TLR4**) recognizes LPS and triggers intracellular signaling, which activates inflammatory factors expression via nuclear factor kappa B (**NF-κB**) and mitogen-activated protein kinase (**MAPK**) signaling (Nyati et al., 2020). Moreover, *S.* Typhimurium-contaminated livestock and poultry-derived products are recognized to be the important sources of zoonotic risk (Kumar et al., 2019). Thus, targeted control of *S.* Typhimurium prevalence in the poultry industry is necessary to effectively reduce the threat of salmonellosis in human.

Currently, various preventive strategies have been used to control *S.* Typhimurium infection in broilers, including vaccination programs and microflora-modulating feed additives in the poultry breeding industry. Among them, antibiotics are the most curative method against *S.* Typhimurium infection (Vandeplas et al., 2010). However, the misuse of antibiotics has inevitably led to the emergence of drugs resistance in *Salmonella*. On the other hand, antibiotic residue also severely affects human health. Therefore, it is imperative to explore effective and safe herbal medicines as alternatives for conventional pharmacotherapy.

Clove oil is a clear pale yellowish volatile liquid extracted from *Syzygium aromaticum*, Myrtaceae, and it is widely used in ambulatory anaesthetization (Correia et al., 2018). Eugenol (C_10_H_12_O_2_; 2-methoxy-4-2-propenyl), the principal active ingredient in clove oil, is well-known for its antibacterial, antifungal, anti-inflammatory, neuroprotective, and antineoplastic properties (Cui et al., 2019; Garabadu and Sharma, 2019; Oroojan, 2020; Sakat et al., 2019; Tandel et al., 2021). In light of that eugenol has acquired a “Generally Recognized as Safe” status from the US Food and Drug Administration, it has extensive applications in pharmaceutical application, health care and cosmetology (Nisar et al., 2021). Indeed, eugenol not only possesses antibacterial activity against *Staphylococcus. aureus, Escherichia coli*, and *Salmonella enterica* in vivo (Muniz et al., 2021; Ruengvisesh et al., 2019), but also relieves LPS-induced intestinal inflammation in mammals (Hui et al., 2020). However, the protective effects of eugenol on *S.* Typhimurium-infected broilers and its underlying molecular mechanism remain unelusive. Hence, the broiler and cell models infected by *S.* Typhimurium were employed to evaluate the protective effects of eugenol, then investigate the potential mechanisms in vivo and in vitro.

## MATERIALS AND METHODS

### Bacterial Strains and Chemical Reagents

*S.* Typhimurium strain SL1344 was used throughout this study, and it was a generous gift from Dr. Yanhong Wang, school of Veterinary Medicine, Yangzhou University, China. SL1344 strain was stored in 30% glycerol at −80°C. The strain was cultured in Luria-Bertani (**LB**) broth under shaking at 37°C to achieve logarithmic growth phases and then was used for further experiments.

Eugenol (≥98.5%) was purchased from Sinopharm Chemical Reagent Beijing Co., Ltd. LB broth was bought from Qingdao Hope Biotechnology Co., Ltd. RNA isolator Total RNA Extraction Reagent, Hiscipt ⅡQRT superMix and ChamQTM SYBR qPCR Master Mix were obtained from Vazyme Biotech Co., Ltd (Nanjing, China). FITC-d-Lys and Rhodamine-conjugated phalloidin were supplied from Xiamen Bioluminor Biotechnology and MedChemExpress, respectively. RIPA buffer (C1053), protease inhibitor (P1265-1) and phosphatase inhibitors (P1260-1) were both bought from Beijing Pleile Gene Technology Co., LTD (Beijing, China). Bicinchoninic acid (**BCA**) assay kit was purchased from Beyotime Institute of Biotechnology (Shanghai, China). Enhanced chemiluminescence (**ECL**) kit and polyvinylidene difluoride (**PVDF**) membranes were bought from Merck Millipore (Billerica, MA). The following antibodies were used to analyze western blotting (**WB**) and immunofluorescence (**IF**). IκBα [4812S, WB (1:1,000)], phospho-IκBα [2859S, WB (1:1,000)], β-actin [4970S, WB (1:1,000)], anti-NF-κB p65 [8242, WB (1:1,000)] and phospho-NF-κB p65 [3033, WB (1:1,000)] were obtained from Cell Signaling Technology (Danvers, MA). ZO-1 [40-2200, WB (1:400), IF (1:100)], claudin-1 [71-7800, WB (1:400), IF (1:100)], occludin [40-4700, WB (1:400), IF (1:200)] were purchased from Thermo Fisher Scientific, Inc. The secondary antibody of WB [111-035-003, (1:5,000)] and IF Alexa Fluor-488 [ab150077, (1:1,000)] were from Jackson ImmunoResearch (West Grove, PA) and Abcam (Cambridge, MA), respectively.

Cultures were routinely incubated in Dulbecco's Modified Eagle Medium (**DMEM**) with glutamine supplemented and 5% (v/v) fetal bovine serum (**FBS**) in 37°C at a humidified 5% (v/v) CO_2_ atmosphere, and used between passages 10 and 20.

### Cell Lines and Culture Conditions

The chicken embryo fibroblast cell line (**DF-1**), mouse-macrophages cell line (**RAW 264.7**), and the rat small intestine cell line (**IEC-6**) were obtained from the cell resource center of Shanghai Institutes for Biological Sciences of the Chinese Academy of Sciences. DF-1, RAW 264.7, and IEC-6 cells were routinely cultured in DMEM supplemented with 10% (v/v) FBS and L-glutamine at 37°C with 5% (v/v) CO_2_. All cells were used for this study at passages 10 to 20. During culture, the medium was changed every 2 d. Moreover, before bacterial adherence and invasion assays, cells were cultured in antibiotic-free mediums for 24 h.

### Bacterial Adherence and Invasion Assays

The effect of the eugenol on the adhesive and invasive ability of *S.* Typhimurium was assessed by employing the gentamicin protection assay. Cells were seeded in a 96-well plate containing the antibiotic-free medium at 37°C, with 5% CO_2_ for 24 h. SL1344 (1 × 10^6^ CFU/mL) were washed twice and re-suspended in DMEM. Then the cells were treated with different concentrations of eugenol (98.5, 191, and 388 μM) and together infected with bacteria (1 × 10^6^ CFU/mL) for 1 h prior to following assays.

In the adhesion assay, the supernatant was discarded, and the cell pellets were washed and lysed with 0.1% TritonX-100 for 20 min, and then collected suspensions. In the invasion assay, medium was removed and cells were incubated for 1 h in the DMEM supplemented with gentamicin (100 μg/mL). Supernatant was discarded and cells were lysed by 0.1% TritonX-100 for 20 min. Finally, the suspensions were diluted with PBS, plated onto LB agar plates at 37°C overnight for bacteria colonies enumerating. All data are shown as means of three independent experiments.

### Confocal Microscopic Examination of Intracellular *S.* Typhimurium

The protective ability of eugenol against *S.* Typhimurium invasion was observed by confocal laser microscopy. The experimental method was performed following the reference (Kuru et al., 2012).

DF-1 cells (1 × 10^5^ per well) were seeded and incubated in 24-well plates with the antibiotic-free medium at 37°C in a 5% CO_2_ atmosphere and pretreated with eugenol. After 16 h cultures, bacteria were washed and centrifuged in sterile phosphate-buffered saline (**PBS**) twice, then resuspended in the antibiotic-free medium with 10 μL FITC-D-Lys. Next, 5 μL of bacteria were added to 24-well plates at 37°C for 1.5 h. Cells were fixed in 4% paraformaldehyde for 15 min, washed with PBS three times and stained with 30 μL phalloidin (1 μg/mL). Finally, the slides were sealed with DAPI-containing anti-fluorescence and visualized under a laser scanning confocal microscope (TCS-SP8, Leica, Germany).

### Animals

All animal experiments had been approved by the committee on the ethics of animal experiments of Yangzhou University, Yangzhou, China and were performed in accordance with the guideline for the care and use of laboratory animals. One-day-old male Yellow Feather Broiler chickens were purchased from the Institute of poultry science, Chinese academy of agriculture science. Broilers were reared according to the literature (Bo et al., 2021; Crhanova et al., 2011).

### Experimental Designs

Seventy-five 8-day-old broilers were separated randomly into control group (Control), SL1344 infection group (SL1344), eugenol pretreatment groups (25, 37.5, and 50 mg/kg, namely SL1344+E-25, SL1344+E-37.5, and SL1344+E-50) with 15 broilers in each group. We repeated three batches of independent experiments with 75 broilers per batch. Eugenol was diluted to the desired concentrations with olive oil (vehicle). A total of 0.2 mL of the corresponding solutions was orally administrated to each 11-day-old chicken once a day for 7 consecutive days in the drug pretreatment groups, while control and SL1344 groups received the same volume of vehicle. SL1344 was cultured in LB broth at 37°C for 22 h, centrifuged, washed twice with aseptic PBS, then concentrated in PBS at a final concentration of 1 × 10^9^ CFU/mL. At 13 d of age, except for the control group, each of the experimental broilers was orally administered with 1 mL bacterial suspension for 3 successive days at a dose of 1 × 10^9^ CFU once a day to establish the *S.* Typhimurium infection model. Broilers were monitored per day for survival rate and body weight. After 12 h fasting, broilers were sacrificed humanely at 18-day-old. The liver, spleen and duodenum were collected for subsequent experiments.

### Organ Bacterial Loads

Organs including heart, liver, spleen, lung, and kidney, were aseptically collected for bacterial load detection. Samples were homogenized in 1 mL sterile PBS at 4°C and 70 Hz for 2 min using a Tissuelyser-24 homogenizer (Jingxin, Shanghai, China). The homogenate solution was diluted serially in PBS. Then, 100 μL of dilutions were uniformly plated on LB agar plates and cultured for 18 h at 37°C.

### Histopathology In Duodenum

Duodenum tissues were washed with PBS and then fixed in 4% paraformaldehyde for 24 h. The fixed tissues were made into paraffin block after a series of procedure such as dehydration with gradient ethanol, clarity with xylene, waxdip and so on, then the paraffin block was sliced into 4-μm sections. Ultimately, the sections were stained with hematoxylin and eosin (**HE**) and examined by microscopy for the histopathological changes.

### Scanning Electron Microscope

Scanning electron microscope (SEM) was used to observe the ultrastructural changes of the duodenum. Samples were rinsed twice in cold PBS and fixed with 2.5% glutaraldehyde solution overnight at 4°C. After being fixed, the tissues were trimmed into 3 mm^3^ and washed three times with precooled PBS, followed by successively dehydration using 30, 50, 70, 85, 95, and 100% alcohol, the tissue blocks were dried in the dryer and sprayed with gold. Then, Geminisem 300 Zeiss field emission scanning electron microscope system (GeminiSEM 300, Carl Zeiss, Germany) was applied to observe and photographing the changes in morphological structure of duodenum.

### Real-Time Fluorescent Quantitative PCR

Total RNA was extracted from duodenum tissues using Trizol reagent (Vazyme, Nanjing, China) following the manufactures’ instructions. Its concentrations and quality were determined by the nanodrop spectrophotometer (Thermo Scientific, Wilmington, DE), and cDNA synthesized using Hiscript II QRT supermax (Vazyme). Real-time quantitative PCR (**RT-qPCR**) was performed with the cfx96 real-time system (Bio-Rad, Hercules, CA,). Templates without cDNA were used as negative controls for each gene in each test. The relative quantitative results were calculated by the 2^−∆ ∆Ct^ method, ∆Ct = CT-CT _actin_, -∆ ∆Ct = -(∆Ct _experimental group_-∆Ct _blank group_). A sample value of the blank group was taken as 1, and the primer sequences for amplifying TNF-α, IL-1β, IL-2, and IL-18 were listed in Table 1. Data are representative of three independent experiments.

**Table 1:** Primers used in the study.

Gene	Forward primer	Reverse primer
*ZO-1*	CAACTGGTGTGGGTTTCTGAA	TCACTACCAGGAGCTGAGAGGTAA
*claudin-1*	TGGAGGATGACCAGGTGAAGA	CGAGCCACTCTGTTGCCATA
*claudin-2*	CCTGCTCACCCTCATTGGAG	GCTGAACTCACTCTTGGGCT
*TLR4*	TGCCATCCCAACCCAACCACAG	ACACCCACTGAGCAGCACCAA
*MyD88*	AGAAGGTGTCGGAGGATGGTG	GGGCTCCAAATGCTGACTGC
*iNOS*	CAGCTGATTGGGTGTGGAT	TTTCTTTGGCCTACGGGTC
*IL-2*	GTGGCTAACTAATCTGCTGTCC	GTAGGGCTTACAGAAAGGATCAA
*TNF-α*	AGATGGGAAGGGAATGAACC	TCAGACATCAAACGCAAAAG
*IL-1β*	GGTCAACATCGCCACCTACA	CATACGAGATGGAAACCAGCAA
*IL-18*	CAGCGTCCAGGTAGAAGATAAG	TCCTCAAAGGCCAAGAACAT
*18sRNA*	AGAAACGGCTACCACATC	GGACTCATTCCAATTACAGG

### Western Blotting Analysis

WB was performed to detect the protein expressions of intestinal tight junction and NF-κB signal pathway. The total proteins of the duodenum were extracted using RIPA buffer containing protease inhibitor (1:50) and phosphatase inhibitors (1:100), and the concentrations of proteins were quantified by BCA kit. Protein lysates were separated in electrophoresis on polyacrylamide gels (8–12%) and transferred onto PVDF membranes. Next, the members were blocked with 5% non-fat milk at room temperature for 3 h and incubated at 4°C overnight with primary antibodies after washing with TBST. This was followed by incubation with suitable HRP-conjugated secondary antibody. Finally, the protein bands were visualized by ECL kit in accordance with the manufacturers’ instructions. The Image J software was used to quantify the protein bands and the targeted protein expressions were normalized to β-actin. The experiment was repeated in triplicate.

### Immunofluorescence Assay

The duodenal paraffin sections were conventionally deparaffinized with xylene, dehydrated through gradient alcohol, and immersed in EDTA antigen repair buffer (PH 8.0) for antigen repair using a microwave oven. These heated slides were cooled down to room temperature for subsequent incubation with primary antibody (claudin-1 dilution) at 4°C overnight. Following washed with cold PBS three times, the slices were incubated with the fluorescent secondary antibody at room temperature for 1 h avoiding light. Next, the sections were washed twice in PBS and stained with DAPI for 15 min. Finally, the expression of claudin-1 was observed and imaged using a confocal laser scanning microscope with a 488 nm excitation (TCS-SP8, Leica, Germany).

### Statistical Analysis

The experimental data were analyzed using SSPS 25.0 statistic software and at least 3 independent replicates. Statistical analysis was determined using a one-way analysis of variance (**ANOVA**) with Duncan the multiple-comparison test. The results were expressed as mean ± SE and represented with GraphPad Prism 8.0 to make the histogram and curves. *P* < 0.05 was considered as significant difference and *P* < 0.01 was extremely significant difference.

## RESULTS

### Eugenol Alleviated the Intestinal Damage of Broilers Challenged by *S.* Typhimurium

**Figure 1:**
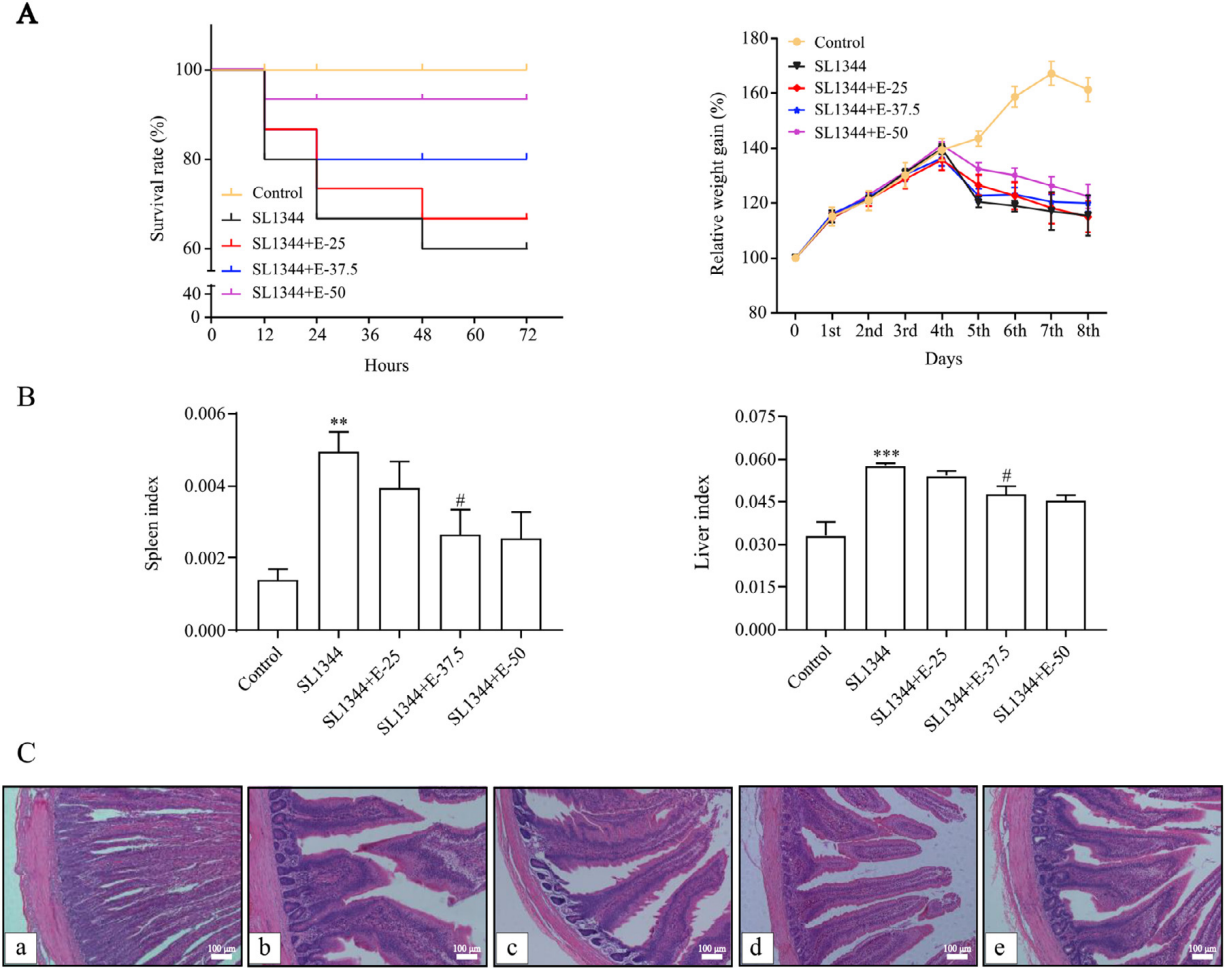
Eugenol relieved the intestinal damage caused by SL1344 in broilers. (A) The relative weight gain rate and survival rate (%). (B) The spleen and liver indexes. (C) Histological changes (100X). (a) Control, (b) SL1344, (c) SL1344+E-25 mg/kg, (d) SL1344+E-37.5 mg/kg, (e) SL1344+E-50 mg/kg. The data are expressed as the Mean ± SE (n = 15). ** *P* < 0.01, *** *P* < 0.001 vs. Control. # *P* < 0.0*5* vs. SL1344 infection.

**Figure 2:**
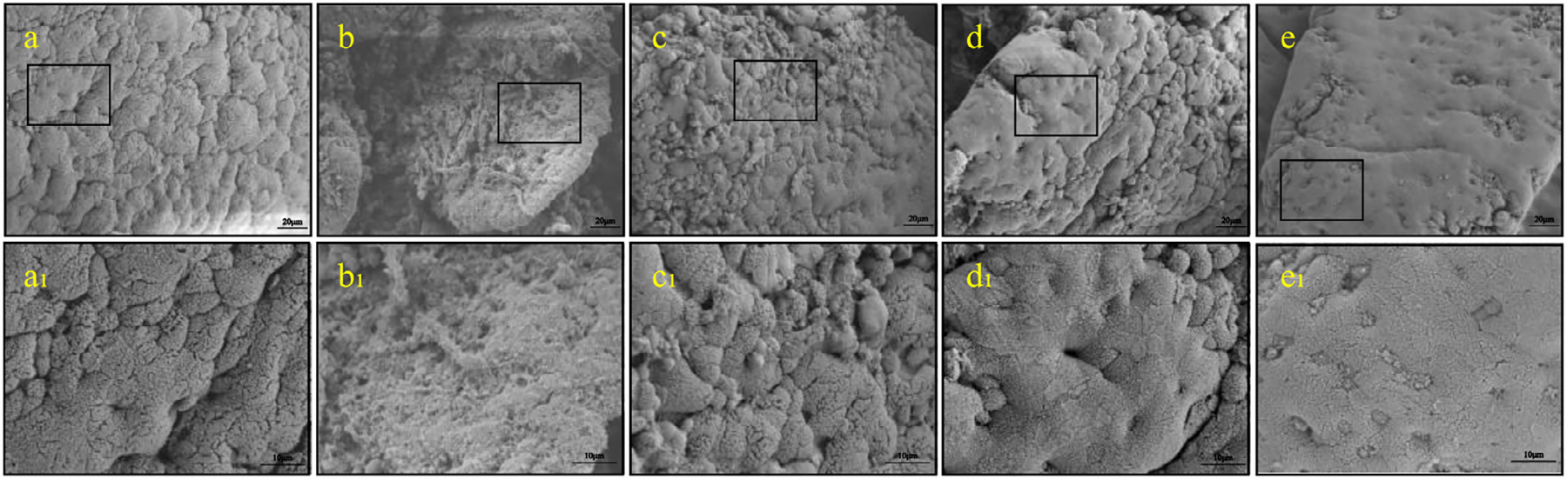
SEM analysis showed eugenol relieved the intestinal ultrastructure changes caused by SL1344 in broilers. (a) and (a_1_) Control, (b) and (b_1_) SL1344, (c) and (c_1_) SL1344+E-25 mg/kg, (d) and (d_1_) SL1344+E-37.5 mg/kg, (e) and (e_1_) SL1344+E-50 mg/kg, the microstructure was observed by SEM (500X and 1500X).

Compared with the control, *S.* Typhimurium infection induced a significant increase in mortality and viscera indexes, accompanying with a reduction of weight. Eugenol relieved the injury of *S.* Typhimurium-infected broilers evidenced by enhancing survival rate, improving relative weight gain and decreasing in viscera indexes as shown in Figures 1A and 1B (*P* < 0.05). Then, we assessed the histological changes of duodenum tissues from each group of broilers (Figure 1C). Pathogen infection caused severe duodenal lesions including the shortening of intestinal villi from the submucosa with edema, inflammatory cells infiltration and congestion, crypt hyperplasia. However, eugenol treatment significantly relieved the above injury, especially the dose of 50 mg/kg. Regarding SEM (Figure 2), it was possible to visualize the disruption of intestinal villi structure, microvilli effacement and bacterial colonization in SL1344 infection. At the same time, 50 mg/kg eugenol had a positive effect to ameliorate duodenum damage.

**Figure 3:**
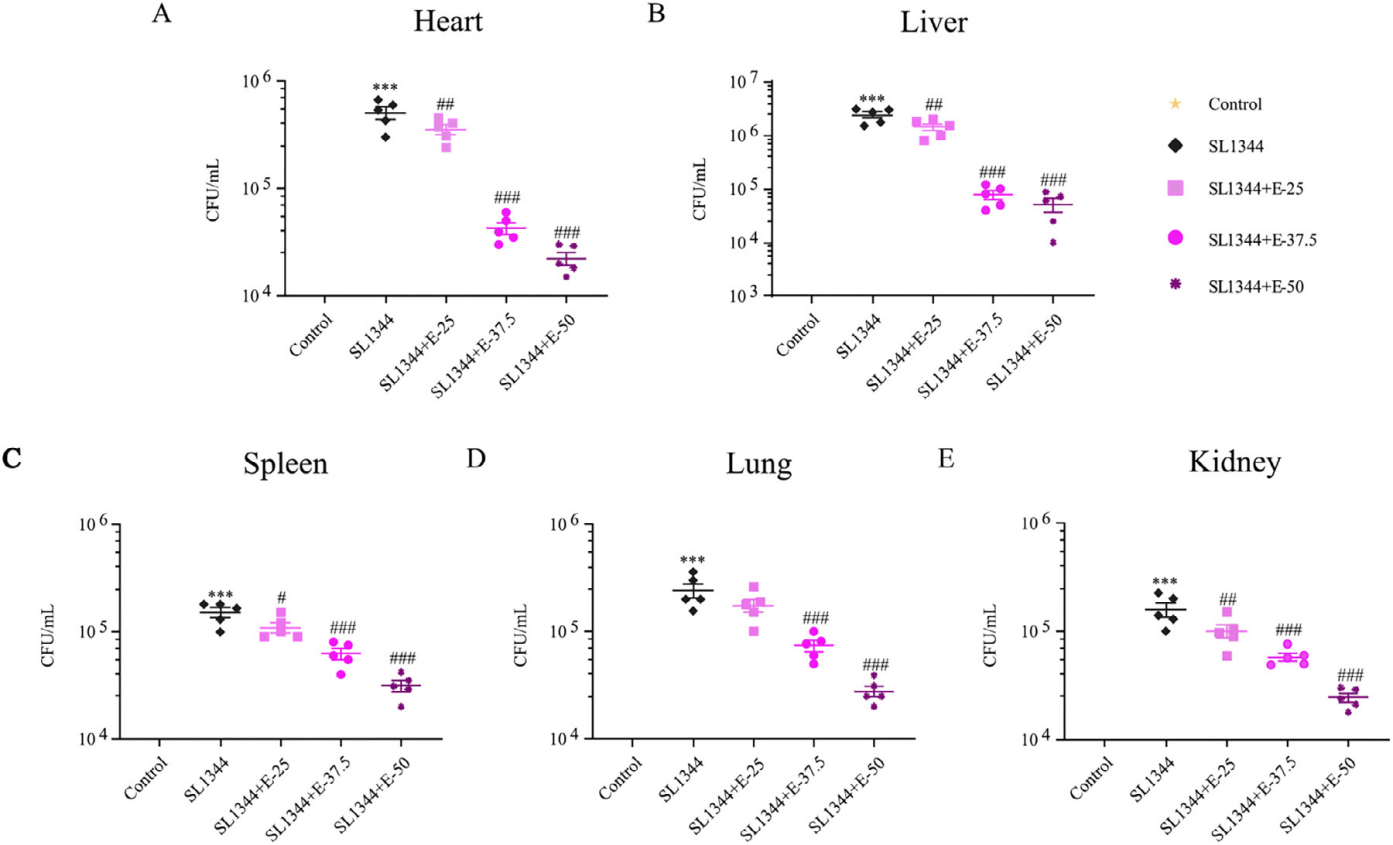
Eugenol pre-treatments reduced the bacterial load in heart (A), liver (B), spleen (C), lung (D), and kidney (E) of post-*Salmonella* infected broilers. The data are expressed as the Mean ± SE (n = 5). *** *P* < 0.001 vs. Control. # *P* < 0.05, ## *P* < 0.01, ### *P* < 0.001 vs. SL1344 infection.

Next, we quantified bacterial loads in heart, liver, spleen, lung and kidney of boilers to explore the inhibiting effect of eugenol on reproduction of *S.* Typhimurium Figure 3. exhibited that eugenol treatment decreased the bacterial organ loads caused by SL1344 infection, especially the high dose (*P* < 0.001).

### Eugenol Suppressed NF-κB Signaling Pathway to Reduce Broilers Intestinal Inflammation Induced by *S.* Typhimurium

**Figure 4:**
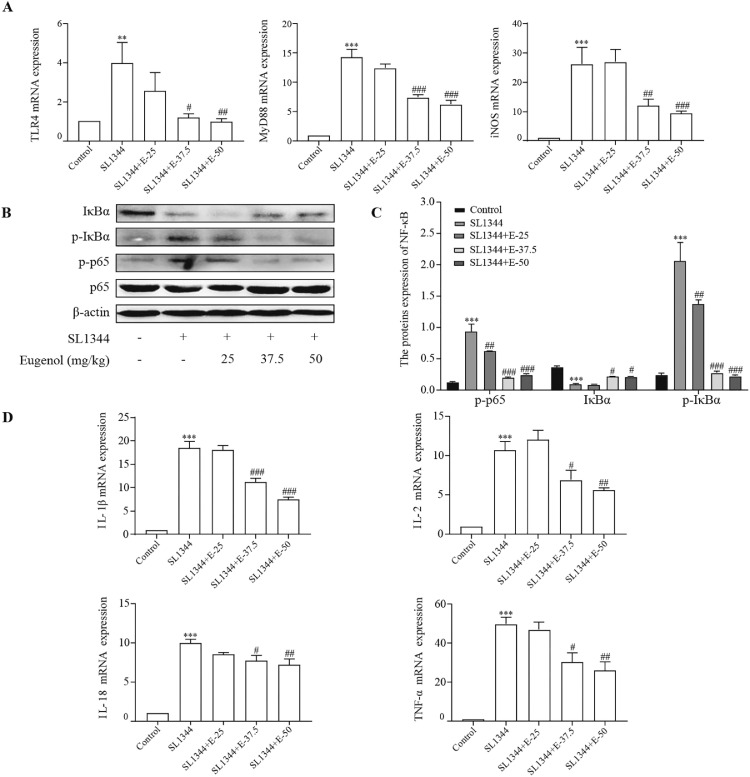
Eugenol pretreatment inhibited the expression of critical mRNA and proteins in NF-κB pathway in duodenal tissue of chickens after *Salmonella* infection. (A) The mRNA expressions of TLR4, MyD88, and iNOS. (B) WB analysis: the protein bands of critical proteins. (C) WB analysis: the quantification of the protein levels by determining the band intensity and normalized to β-actin band levels. (D) mRNA expressions of inflammatory factors (IL-1β, IL-2, IL-18, and TNF-α). All experiments were repeated in triplicates. The data are expressed as the Mean ± SE (n = 15). **P < 0.01, ***P < 0.001 vs. Control. #*P* < 0.05, ##*P* < 0.01, ###*P* < 0.001 vs. SL1344 infection.

TLR4, as an innate immune receptor, can activate NF-κB via triggering the MyD88-independent pathway. To elucidate the protective mechanism of eugenol on *S.* Typhimurium-infected intestinal inflammation in broilers, we detected the expressions of key mRNA and proteins in NF-κB signaling pathway, and its downstream inflammatory cytokines by RT-qPCR and WB. Compared with control, *S.* Typhimurium infection significantly enhanced the mRNA levels of TLR4 and MyD88, meanwhile augmented the phosphorylation of p65 and IκBα in the duodenum of broilers (Figures 4A–4C). In contrast, both concentrations of eugenol treatments (37.5 mg/kg and 50 mg/kg) decreased the mRNA expressions of TLR4 and MyD88, suppressed the degradation of IκBα degradation and overexpression of p-IκBα, then blocked the nuclear translocation of p65 (*P* < 0.001). iNOS and inflammatory factors play an essential role in inflammation. Similarly, the expressions of inflammation-related genes shown in Figure 4D, including TNF-α, IL-1β, IL-2, and IL-18, were downregulated in eugenol-treated (37.5 mg/kg and 50 mg/kg) groups (*P* < 0.001). The tendency of iNOS expression was consistent with the above results. These results indicated that eugenol could inhibit *S.* Typhimurium-infected activation of NF-κB signaling pathway and decline the secretions of pro-inflammatory cytokines. This may be a potential mechanism by which eugenol regulated intestinal inflammation and host immune response infected by *S.* Typhimurium in broilers.

### Eugenol Improved *S.* Typhimurium-Infected Intestinal Barrier Dysfunction Via Upregulating the Expressions of TJs Proteins

**Figure 5:**
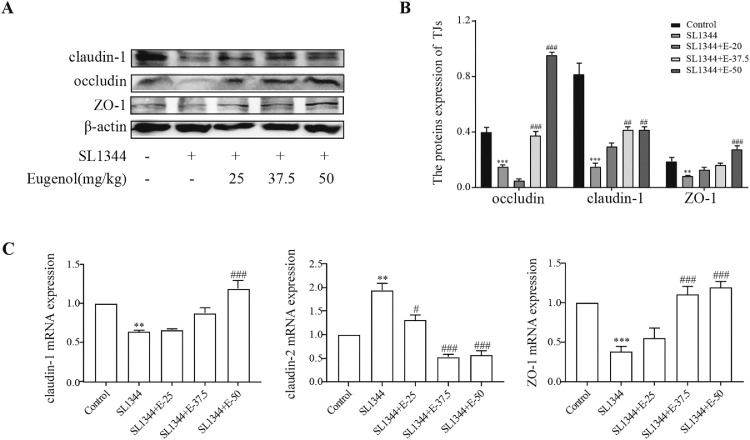
Eugenol improved the expressions of the duodenal tight junction proteins ZO-1, claudin-1 and occludin in *Salmonella*-infected broilers. (A) WB analysis: the protein bands of critical proteins. (B) WB analysis: the quantification of the protein levels by determining the band intensity and normalized to β-actin band levels. (C) The mRNA expressions of tight junctions (ZO-1, claudin-1 and claudin-2). All experiments were repeated in triplicates. The data are expressed as the Mean ± SE (n = 15). **P < 0.01, *** *P* < 0.001 vs. Control. # *P* < 0.05, ## *P* < 0.01, ### *P* < 0.001 vs. SL1344 infection.

**Figure 6:**
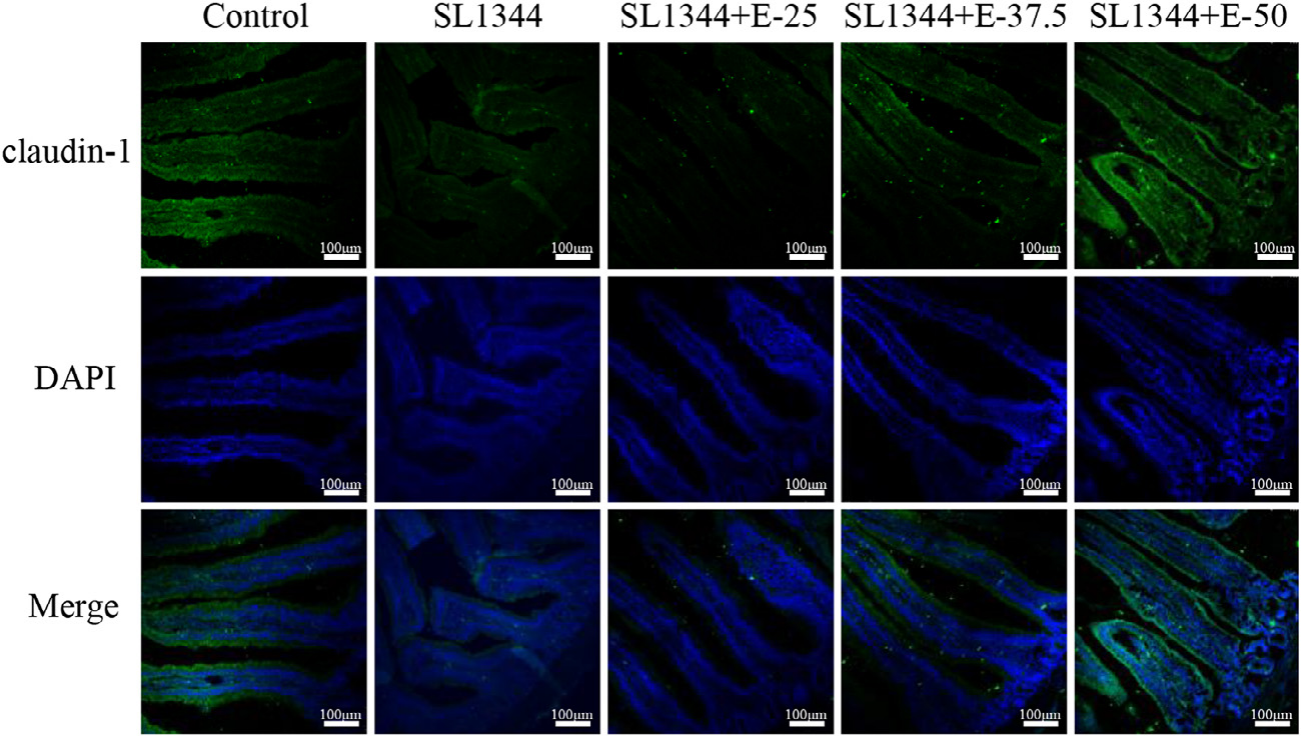
Eugenol improved the expression of tight junction protein claudin-1 in the duodenum of *Salmonella*-infected broilers by immunofluorescence. Green fluorescence represents claudin-1, and blue (DAPI dye) represents the nuclei.

Enteropathogens contribute to the disruption of the intestinal barrier function. Tight junction proteins (**TJs**) form an important intercellular adhesion complex and constitute the apical part of the intestinal epithelium cells, which play a vital role in maintaining the gastrointestinal mucosal barrier and intestinal homeostasis. Thus, we examined the representative proteins level. The WB analysis (Figures 5A and 5B) visualized that the expressions of TJs proteins, including ZO-1, claudin-1, and occludin, in broiler duodenum from the SL1344 group were significantly decreased (*P* < 0.01) compared to the control. However, eugenol pretreatments (SL1344+E-37.5 and SL1344+E-50) upregulated the expressions of ZO-1, claudin-1 and occludin. Meanwhile, the mRNA expressions were consistent with the protein expressions (Figure 5C). We also detected the claudin-1 expression and localization by immunofluorescence. *S.* Typhimurium infection greatly diminished the fluorescence at a site of claudin-1 around the cellular borders, while eugenol pretreatments inverted this trend, among them, the *S. T*+E-50 group showed relatively complete expressions (Figure 6). These results indicated that eugenol effectively maintained the expressions of TJs.

### Eugenol Inhibited the Adhesion and Invasion of *S.* Typhimurium in Vitro

**Figure 7:**
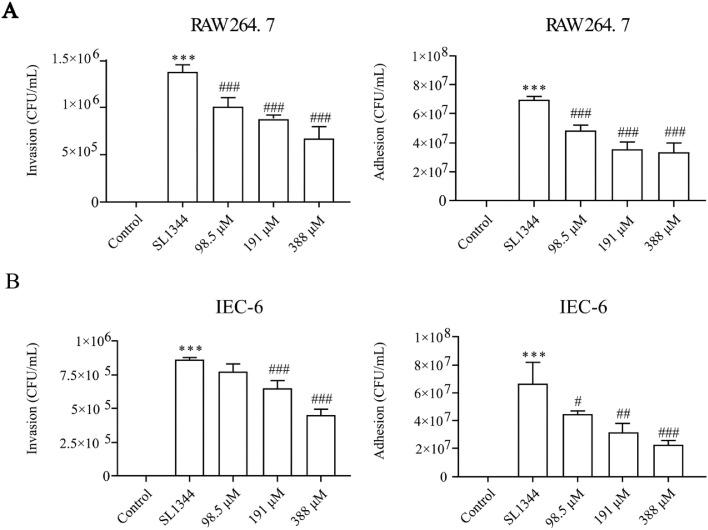
Eugenol remarkably inhibited the adhesion and invasion of *Salmonella* to cells in vitro. (A) SL1344 adhesion and invasion assays on RAW 264.7 macrophages. (B) SL1344 adhesion and invasion assays on IEC-6 cells. The data are expressed as the Mean ± SE (n = 3). *** *P* < 0.001 vs. the Control. # *P* < 0.0*5*, ## *P* < 0.01*,* ### *P* < 0.001 vs. the SL1344 infection.

*S.* Typhimurium could adhere to and invade the intestinal mucosa. As shown in Figures 7A and 7B, eugenol reduced the bacterial adhesion and invasion to IEC-6 cells and RAW 264.7 cells (*P* < 0.05), especially the concentration of 388 μM.

**Figure 8:**
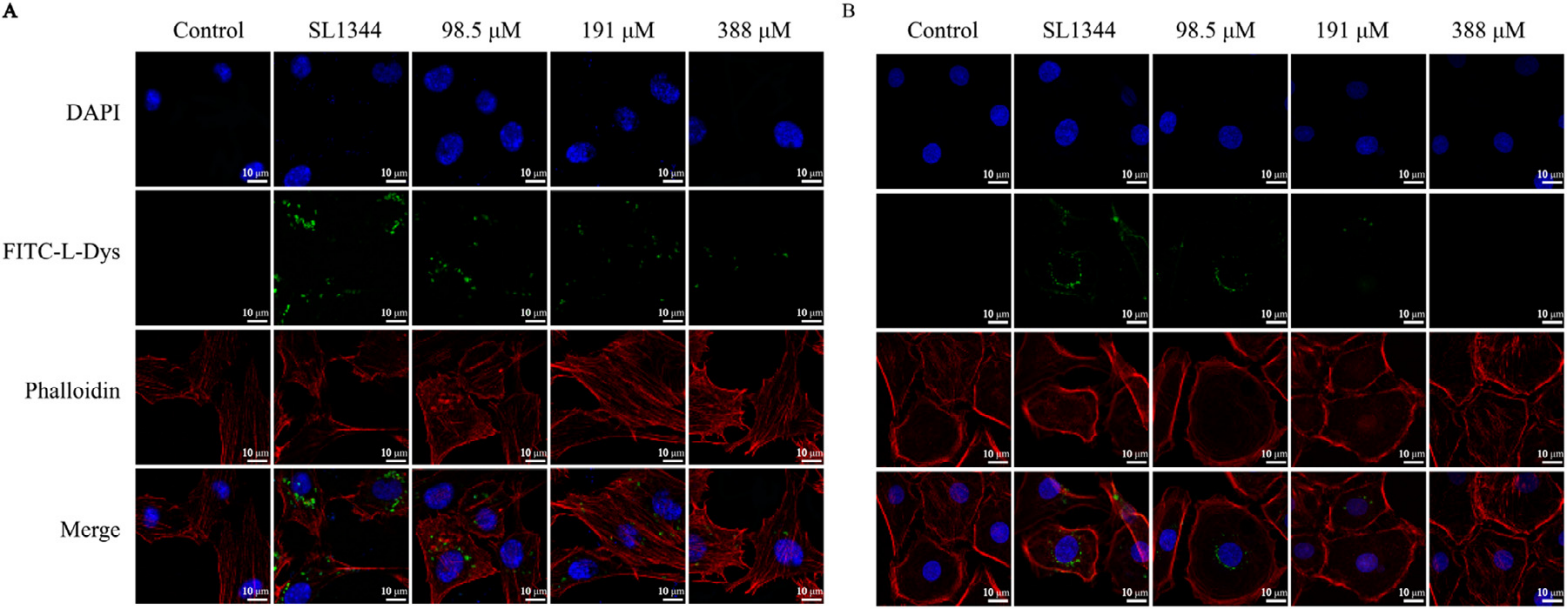
Confocal images of cells treated with various concentrations of eugenol and infected with SL1344. (A) DF-1 cells. (B) IEC-6 cells. Phalloidin was visualized as red, SL1344 as green, and DAPI as blue.

Phalloidin staining showed a fusiform structure characteristic of DF-1 and IEC-6 cells, FITC-d-Lys staining showed the quantity and location of SL1344. After 4 h infection, a lot of bacteria were localized in the intracellular compartment of DF-1 cells (Figure 8A), and the cell morphology appeared incomplete with obscure boundaries. Eugenol exposure decreased bacteria count in DF-1 cells. The similar trend was also be observed in IEC-6 cells (Figure 8B).

## DISCUSSION

*S.* Typhimurium mainly damage the gastrointestinal system of avian, which is a highly infectious disease that can result in high mortality in poultry industry and pose a serious threat to safe food for public human health. Due to the indiscriminate use of antibiotics in agriculture and animal husbandry, conventional therapies induce drug resistance or residue accumulation, and side effects on intestinal flora. Thus, the new and safe treatment for controlling *Salmonella* is imminent. In this study, we preliminarily investigated the protective efficacy of eugenol on broilers challenged by *S.* Typhimurium and further revealed its potential mechanisms in vivo and in vitro.

*Salmonella*’s particular invasion mechanism and virulence factors are vital for its infecting and surviving in vivo. Difference with other pathogens, *Salmonella* utilizes the zipper and trigger mechanisms to invade host cells (Dokladny et al., 2016). *Salmonella* pathogenicity island-1 (**SPI-1**) of Type III secretion system (**T3SS**) is closely linked with bacterial invasion to host cells through inducing membrane ruffling and disrupting actin polymerization. In addition, the SPI-2 maintains systemic virulence and facilitates intracellular survival (Parikh et al., 2019). All along, eugenol represents an broad-spectrum excellent antibacterial agent. It has a stronger antimicrobial effect on *S*. Typhimurium (SL1344) with a MIC of 0.0125% (v/v) (Zhao et al., 2022). Our previous study has proved eugenol exposure in vitro inhibits the expressions of T3SS and Type I fimbriae (**TIF**) virulence genes in *S.* Typhimurium, then resulting in a significantly reduction of pathogenicity to chickens (Zhao et al., 2022). It has been reported that ginsenoside Rg3 significantly reduced in *S.* Typhimurium strains adhesion to RAW 246.7 cells (Mechesso et al., 2021). In this study, our results showed that eugenol decreased the adhesion and invasion of *S.* Typhimurium to IEC-6 and RAW264.7 cells. Moreover, eugenol inhibited bacterial intracellular multiplication of DF-1 and IEC-6 cells. Therefore, eugenol could reduce adhesion and invasion abilities of *S.* Typhimurium to cells through suppressing the expressions of TIF-related adhesion virulence factors.

The small intestine is a strong immune barrier against potential pathogens (Bazzoni and Dejana, 2004). The first line of defense against the invasion of pathogens is the mucus layer in the intestinal epithelial surface, which functions as a physical protective barrier to avoid pathogens (Bazzoni and Dejana, 2004). The intestinal barrier, which is formed by the epithelia cells and TJs, contributes to the protection and regulation of intestinal homeostasis (Bazzoni and Dejana, 2004). TJs are assemblies of multiple transmembrane proteins (such as claudins, occludin), and peripheral cytoplasmic scaffold proteins (zonula occludens, **ZO-1**) (Dokladny et al., 2016). Destruction of TJs influences the integrity and permeability of the intestinal barrier, which results in inflammatory responses (Parikh et al., 2019). Indeed, increasing evidence indicated that maintaining TJs proteins expressions could regulate barrier integrity and reduce pathogen-induced intestinal damage in vivo or in vitro model (;Chen et al., 2021 Dong et al., 2020; Liu et al., 2022; Moral-Anter et al., 2020). In addition, eugenol, as phytonutrient diet supplementation, can strengthen the mucosal barrier of mice to defend against invading pathogens (Wlodarska et al., 2015). To elucidate the mechanism of eugenol in preventing intestinal barrier disruption caused by SL1344, we detected the changes in the expressions of ZO-1, claudin-1, claudin-2, and occludin. The results illustrated that the proteins and mRNA expressions of TJs proteins were downregulated after *S.* Typhimurium infection. However, eugenol pretreatments reversed this trend. Consistent with our results, eugenol is beneficial to maintain barrier function by means of upregulating the relative mRNA abundance of ZO-1, and CLDN-1 (Hui et al., 2020). In summary, eugenol could maintain the intestinal barrier function and preserve intestinal homeostasis by stabilizing the TJs proteins.

Epithelial barrier destruction could trigger excessive host inflammatory and immune responses by *S.* Typhimurium invading to the intestinal mucosa. Overexpression of proinflammatory cytokines are the major biomarkers of exacerbated intestinal inflammatory response. *Salmonella* infection causes a strong proinflammatory response (Ktsoyan et al., 2015), and it is accompanied by an influx of inflammatory cells (Antunes et al., 2011). IL-2, an important cytokine secreted by type 1 T-helper cells, plays a crucial role in the functional activation of the cells of the innate immune response (Ibrahim et al., 2021). TNF-α, as the downstream of NF-κB, mainly mediates inflammation, immunity and apoptosis (Li et al., 2017). IL-1β and IL-18 are primarily responsible for triggering the intestinal inflammatory response characteristic of salmonellosis (Dos Reis & Horn, 2010). iNOS is widely regarded as the key inflammatory protein expressed at inflammatory sites that participate in the regulation of intestinal inflammation and oxidative stress by producing excessive NO (Fan et al., 2020). NO could stimulate the secretion of cytokines, such as TNF-α and IL-1β, further activate iNOS (Wallace and Miller, 2000). Therefore, it is important that anti-inflammatory drugs target the production of proinflammatory mediators to protect the organism from pathogens infection. In the present study, eugenol obviously attenuated the intestinal inflammation by downregulating the expressions of TLR4, MyD88, iNOS, p-p65, p-IκBα, and inflammatory factors (TNF-α, IL-1β, IL-2, IL-18). Therefore, these results indicated that eugenol could serve as a candidate drug for *S.* Typhimurium infection with underlying mechanisms that affected the NF-κB signaling pathway and stabilized intestinal barrier integrity. Consistent with our results, decrease of proinflammatory cytokines can relieve intestinal inflammation in *S.* Typhimurium-induced diarrhea mice (Zhang et al., 2017, 2020)

Accordingly, we further studied the anti-inflammatory molecular mechanisms of eugenol in *S.* Typhimurium challenged broilers. Emerging evidence suggested that the productions of proinflammatory cytokines are strongly associated with TLR4/MyD88/NF-κB signaling pathway (Zhang et al., 2017). LPS, as the prototypical pathogen-associated molecular pattern (**PAMP**) of Gram-negative bacteria including *Salmonella*, was recognized by host innate immunity pattern recognition receptors (**PRR**). Among these, TLR4 is a major receptor for LPS recognition (Yang et al., 2019). LPS could activate TLR4, resulting in activation of numerous inflammatory pathways, such as NF-κB (Sugimoto et al., 2015). Next, stimulation of TLR4 recruits the adaptor protein myeloid differentiation factor 88 (**MyD88**), which activates the downstream signaling, such as NF-κB and subsequently causes the production of proinflammatory cytokines (Girón et al., 2010). NF-κB consists in homodimer and heterodimers of the Rel/ NF-κB family proteins (p50, p52, p65, RelB, and c-Rel), among them NF-κB p65 is the most commonly explored (Coupienne et al., 2011). The IκB kinase (**IKK**)-mediated phosphorylation of the IκBα results in nuclear translocation of the NF-κB p65, which is an indicator of NF-κB pathway activation; then, the transcription of various proinflammatory factors takes place in the nucleus (Chung et al., 2008). In fact, the intestinal inflammation and barrier disruption were attenuated in TLR4, MyD88, and IKK genes deficient mice (Liu et al., 2009; Wang et al., 2020). In support of our findings, many in vivo researches confirmed that inhibition of the TLR4 and NF-κB-associated key proteins expressions to alleviate inflammatory response (Chen et al., 2021b; Dong et al., 2020). Our results showed that *S.* Typhimurium infection stimulated the higher expressions of TLR4, MyD88, p-p65, and p-IκBα in the broilers. However, eugenol treatments could downregulate the expressions of above factors, proving the inhibitory effect of eugenol on *S.* Typhimurium-induced inflammatory response. Therefore, based on the above analysis, it can be inferred that the inhibiting NF-κB signaling pathway and improving disrupted epithelial barriers contribute to the protective effects of eugenol on *S.* Typhimurium-infected intestinal inflammation and injury.

## CONCLUSIONS

Taken together, our results confirmed that eugenol pretreatment protected broilers from *S.* Typhimurium-challenged intestinal inflammation and injury through blocking NF-κB signaling pathway, repressing the secretion of inflammatory factors, and then improving intestinal barrier function. Moreover, eugenol could inhibit bacterial adhesion and invasion to IEC-6, RAW264.7, and DF-1 cells in vitro. These findings provided a novel insight into the potential prophylactic application of eugenol to avian bacterial diseases.
